# Parenting and Sibling Relationships in Family with Disruptive Behavior Disorders. Are Non-Clinical Siblings More Vulnerable for Emotional and Behavioral Problems?

**DOI:** 10.3390/brainsci11101308

**Published:** 2021-10-01

**Authors:** Martina Smorti, Emanuela Inguaggiato, Lara Vezzosi, Annarita Milone

**Affiliations:** 1 Department of Surgical, Medical and Molecular Pathology and Critical Care Medicine, University of Pisa, 56126 Pisa, Italy; lara.vezzosi2@gmail.com; 2 Department of Child Psychiatry and Psychopharmacology, IRCCS Stella Maris Foundation, 56018 Pisa, Italy; emanuela.inguaggiato@fsm.unipi.it (E.I.); annarita.milone@fsm.unipi.it (A.M.)

**Keywords:** disruptive behavior disorders, parenting style, sibling relationship, emotional and behavioral problems

## Abstract

Disruptive Behavior Disorders (DBD) are the most common mental health disorders in the school-aged child population. Although harsh parenting is a key risk factor in the shaping of DBD, studies neglect the presence of siblings and differential parenting. This study aims to compare: (1) parenting style and sibling relationship in sibling dyads of clinical families, composed of a DBD child and a non-clinical sibling, with control families composed of two non-clinical siblings; (2) parenting style, sibling relationship, and emotional and behavioral problems in DBD child, non-clinical sibling, and non-clinical child of control group. Sixty-one families (composed of mother and sibling dyads), divided into clinical (*n* = 27) and control (*n* = 34) groups, completed the APQ, SRI, and CBCL questionnaires. Results indicated differential parenting in clinical families, compared to control group families, with higher negative parenting toward the DBD child than the sibling; no difference emerged in sibling relationship within sibling dyads (clinical vs. control). Finally, externalizing and internalizing problems were higher in DBD children and their siblings, compared to control, indicating DBD sibling psychopathology vulnerability. Findings suggest inclusion of siblings in the clinical assessment and rehabilitative intervention of DBD children, given that the promotion of positive parenting could improve mental health in the offspring.

## 1. Introduction

Disruptive Behavior Disorders (DBDs) including Oppositional Defiant Disorder (ODD) and Conduct Disorder (CD), are a challenging mental health issue and represent the most common mental health reason for referral for school-aged youths [1,2,3]. DBDs, according to Diagnostic and Statistical Manual of Mental Disorders, Fifth Edition, are a cluster of disorders defined by the presence of a persistent pattern of negative, defiant, aggressive, rule-breaking or disruptive behaviors, such as a repetitive and persistent pattern of behavior that violates the basic rights of others or violates major age-appropriate societal rules or norms [4]. These characteristics often emerge early in development and persist into adolescence and adulthood, leading to widespread difficulties, causing clinically significant impairment in the youth’s social, academic, familial, or personal functioning [4,5,6,7].

DBD children and adolescents are at risk of developing many severe psychopathological outcomes in adolescence and as adults (mood and bipolar disorders, psychopathy, and antisocial personality), with worsening prognosis and social costs [8,9,10]. The risk of later psychopathology (ADHD, mood disorders, substance use, suicidality), and poor overall functioning, is increased in DBDs in the presence of deficits of emotion regulation, characterized by lack of temper control, affective lability, mood instability, and emotional overreaction [11,12,13,14,15,16].

DBD is a multi-determined condition involving both biological and environmental factors; in fact, genetic, temperamental, environmental, social, and family factors, and the interaction between “nature” and “nurture”, seem to influence developmental risk for DBD in children [17,18,19,20,21]. In the text the term child/children will be used to refer to offspring whether these are children or adolescents.

Regarding biological factors, several studies show moderate influence of these factors in the etiology of DBD, especially in the early-onset form, pervasive conduct disorder, and in relation to callous-unemotional traits. Molecular genetic studies, also conducted in twins, have shown associations between genetic variants of genes of the dopaminergic and serotonergic systems and DBD [22,23].

Previous studies have indicated that parenting styles are key factors in shaping DBD in children. Specifically, the use of harsh parenting, which includes coercive, controlling, and punitive methods, the use of verbal and physical aggression [24,25,26,27], combined with low warmth and poor positive parenting [28,29,30], are associated with DBDs as well as more general externalizing disorders in children and adolescents [31,32]. Despite the absolute level of negative parenting, children who report harsher and less supportive parenting tend to manifest more externalizing behaviors compared to their siblings [33,34,35,36,37,38]. Studies on family risk factors have mainly focused on the effect of specific parenting styles on DBD child adjustment [39,40], neglecting the presence of siblings. A perspective that takes into consideration parenting style on sibling dyads may allow the identification of risk and protective factors for the psychological adjustment of offspring in the same family, to better address preventive intervention [40].

Despite the large amount of literature on the effects of parental practices that are directed unequally among siblings (differential parenting [41]) and on externalizing behaviors in children from the same family, less is known about the effect of differential parenting on externalizing behaviors reported by children and adolescents in families with a DBD child. It must be noted that DBDs present specific features that increase the risk for several negative outcomes, such as acts of aggression, violence, and risk taking, in addition to the risk for psychopathology [42,43,44].

This study aimed to explore the impact of both parenting style and differential parenting on emotional and behavioral problems of DBD siblings and non-clinical siblings who shared the same environment. In Italy, where we conducted this study, mothers still maintain the central role of main caregivers of children below 18 years, despite the recent increased involvement of fathers in childcare activities [45]. In addition to the task of child-rearing, Italian mothers also have the role of providing guidance and the transmission of norms [46]. Moreover, the quality of the adolescent’s relationship with the mother has a much stronger effect on adolescent risk behavior [47,48] compared to the paternal relationship. Therefore, in the current study we focus on maternal parenting.

This study also aimed to compare maternal parenting styles in families in which one sibling was diagnosed with DBD, while the other did not receive any psychiatric diagnoses, and control families with two non-clinical siblings. This comparison was aimed to identify if parenting styles in clinical families were characterized by more harsh and differential parenting towards clinical than non-clinical children/adolescents compared to control families. We expected higher differential parenting in families of the clinical sample compared to control families, with more negative parenting reported towards the DBD child compared to sibling.

Moreover, we aimed to compare maternal parenting style and emotional and behavioral problems reported in DBD siblings (DBDs), non-clinical siblings (S-DBDs), and non-clinical siblings of control families (CONTR), for early evaluation whether non-clinical siblings in families with DBD would present a higher risk for harsh and negative parenting, externalizing problems, and emotion dysregulation, compared to families without DBD. We expected that non-clinical siblings in families with DBD would report a lower level of negative parenting and externalizing problems compared to their DBD siblings, but higher compared to that reported in control group families.

Another aspect that has been largely neglected in the literature on DBDs until now is the role of sibling relationship quality. Previous studies, using a dyadic perspective, have shown that sibling relationship is linked to differential parenting, and that differential parenting has negative effects on sibling relationship. In fact, although a certain degree of differential parenting is normal and depends on a child’s characteristics, such as age, gender, and temperament [49], differential treatment is linked with more negative sibling relationship as greater conflict and less affection between siblings [49,50], and as more externalizing behaviors, aggression, depressed mood, anxiety, and low self-esteem [40,50,51]. Moving from this consideration, we expected higher levels of conflict reported by siblings of the clinical group compared to the control group. Higher levels of conflict were expected based on parental differential treatment and on characteristics of the DBDs; in fact, typical patterns of externalizing disorders, such as negative, defiant, aggressive, rule-breaking, or disruptive behaviors, can also be implemented in families.

## 2. Materials and Methods

### 2.1. Sample

Our sample included two groups of families (clinical and control) composed of: (a) two biological siblings aged 6–16 years; and (b) their mother. In line with other studies, in which age spacing of four years between siblings is recognized as a strong correlate of differential parenting [52,53], only sibling dyads with age spacing of four years or less were considered for this study.

To be included in this study, participants (mother and offspring) had a good comprehension of the Italian language, gave consent to participate in the study, and completed the battery of questionnaires.

Additional inclusion criterion for the clinical-family sample were: (a) the presence of a child with a diagnosis of DBD according to DSM 5 criteria with Full IQ Scale greater than 85 and no comorbidity with Autism Specter Disorder diagnosis; (b) a sibling without any clinical diagnosis.

For the control group, an additional inclusion criterion was: pairs of siblings in which neither have been submitted to a clinical consultation.

The clinical group was enrolled in the Department of Child and Adolescent Psychiatry of a third level hospital (FSM Stella Maris Foundation in Pisa), which provides care to patients from all over Italy. Recruitment took place in the clinic for the diagnosis and treatment of behavioral disorders in developmental age called “Beyond the clouds”. Families were consecutively recruited during the diagnosis process by the person responsible for this study. Following the diagnosis of DBD, an evidence-based multimodal treatment was offered to the clinical families.

Diagnoses of DBD, based on DSM-5 diagnostic criteria, were made by using historical information, a structured clinical interview, and the Italian version of the Schedule for Affective Disorders and Schizophrenia for School-Age Children—Present and Lifetime Version (K-SADS-PL) [54], administered by child psychiatry trainees under the supervision of the senior child psychiatrist. Diagnoses were definitively confirmed by consensus of a multidisciplinary board.

The Italian version of the Wechsler Intelligence Scale for Children—Fourth Edition (WISC-IV) [55], was administered to all patients to assess IQ.

The control group was recruited during leisure and sports activities in the metropolitan area of Central Italy. Participants (parents and children) were randomly selected among participants of leisure activities and approached when they finished. They were informed about the goals of the survey, and were told they had the option to drop out at any given moment if they wished to do so. Written informed consent from parents was obtained for all participants.

Of the 66 families who were contacted for this study that met inclusion criteria, three (two belonging to control group and one to clinical group) refused to participate due to lack of time. Two families (belonging to clinical group) could not be included in the final sample due to incomplete data (missing questionnaire about one sibling).

The final sample of the study was made up of 61 families: (a) 61 mothers, and (b) 61 couples of siblings, for a total of 122 children (87 males and 43 females), aged between 6 and 15.7 years (Mage = 9.8; SD = 2.1). The sample was divided into two groups, according to inclusion criteria.

The clinical group was composed of 27 families: (a) 27 mothers, and (b) 27 couples of siblings, for a total of 54 children (40 males, 14 females), aged between 6 and 14.8 (Mage = 9.9; SD = 2.0). For each pair of siblings, we labelled DBD the target child who reported the clinical diagnosis, and S-DBD the older sibling of the DBD, closest in age, with no clinical diagnosis.

For the control group, we considered a child comparable in age to DBD, labelled CONTR-1, and an older sibling, labelled CONTR-2. The control group was composed of 34 families: (a) 34 mothers, and (b) 34 pairs of siblings, for a total of 68 children (41 males, 27 females), aged between 6 and 15.7 (Mage = 9.8; SD 2.2). This sample has not been used in previous studies.

The investigator responsible for the study informed participants about the main study aims, specified that anonymity was guaranteed, participation was voluntary, they could withdraw from participation in any moment, and no monetary reward was given. A signed statement attesting to informed consent for study participation was obtained from all subjects. After the informed consent was signed, according to the study protocol, the mother filled in the questionnaire about parenting style and psychological problems for each child/adolescent, and each child/adolescent completed a specific questionnaire to evaluate the perception of the quality of the sibling relationship. Participants completed all questionnaires, and no missing data were reported.

The study conformed to the Declaration of Helsinki. The Regional Ethics Committee for Clinical Trials of Tuscany, Pediatric Ethics Committee section, approved the study (ethical approval code: GENCU/03/2019). Recruitment took place from April 2019 to October 2020 and lasted 18 months.

### 2.2. Measures

Mothers completed the Child Behavior Check List (CBCL) parent report form and Alabama Parenting Questionnaire (APQ) for each child. Each child/adolescent completed a questionnaire that evaluates the perception of the quality of sibling relationship: Sibling Relationship Inventory (SRI).

Socio-demographic, clinical, and socio-economic status were collected from the mother using an ad hoc questionnaire.

The CBCL [56,57,58] was used to explore and record emotional and behavioral problems and skills in children aged 6–18 years. It is a 118-item scale, asking parents how often his/her child engages in problematic behaviors (i.e., item 28 “Breaks rules at home, school, or elsewhere”). According to questionnaire norms [56,57,58], CBCL presents 8 different syndrome scales, 6 different DSM-Oriented scales, a total problem score, and two broad-band scores designated as internalizing problems and externalizing problems. The reliability coefficients (Cronbach’s alpha) were 0.82, 0.81, and 0.82, respectively.

The CBCL calculates a specific profile correlated to poor self-regulation in children and adolescents by summing the T Score of CBCL scale Anxious/Depressed, Attention Problems, and Aggression Behaviors. Emotional Self-Regulation (DESR) profile is given to those who present a summed score > 180 but < 210 and Dysregulation Profile (DP) as those who present a summed score ≥ 210 [59,60]. The DESR profile has been related to maladaptive behaviors in response to frustration or negative emotions, impulsivity, elevated irritability and anger, and high rates of anxiety and disruptive behavior disorders [61]. The DP has demonstrated usefulness as a marker of dysregulation and psychopathology severity in children and adolescents, and has been associated with severe psychopathology, self-harm and suicidal behaviors [59,62], and substance use disorders [63].

The APQ [64,65], was used to assess parenting practices and quality of the parent-child relationship. The APQ includes 42 items requiring parents to indicate the frequency with which they implement the parenting practices using a 5-point Likert scale, ranging from 1 (never) to 5 (always). According to Italian validation of the APQ [65], the parenting domains measured by this instrument are: (1) positive parenting (PP), comprising parental involvement and positive parenting (i.e., item 2: “You let your child know when he/she is doing a good job with something”); (2) negative parenting (NP), comprising poor monitoring/supervision, inconsistent discipline (i.e., item 24: “You get so busy that you forget where your child is and what he/she is doing”); (3) corporal punishment (CP) (i.e., item 33: “You spank your child with your hand when he/she has done something wrong”). The subscale score is derived by a sum of the items. Higher scores indicate adequate parenting practices for the positive scale, inadequate parenting practices for the negative scale, and corporal punishment. In this study, the Cronbach α coefficients were 0.73 for Positive Parenting, 0.68 for Negative Parenting, and 0.74 for Corporal Punishment.

The SRI [66,67] was used to assess the quality of sibling relationship. This is a 17-item questionnaire developed to assess the child’s perception of own behavior and own feelings towards sibling. The SRI assesses 3 qualitative dimensions of sibling relationship: (1) Affection, referring to behaviors of support, help, sharing and admiration between siblings (i.e., item 1: “Do you happen to take care of your brother or sister or deal with him/her if there are no adults?”), (2) Conflict, defined as disagreement and quarrels (i.e., item 3: “Do you happen to be very angry with your brother/sister?”); (3) Rivalry, which is the perception that children have of differential treatment from their parents (i.e., item 11: “Do you also think that your mother treats your brother/sister better than you?”). Individuals are required to indicate how often they experience content of item on a 5-point Likert scale ranging from 1 (never) to 5 (always). Total score is obtained summing the scores for the three dimensions. The questionnaire was proposed to each child as an interview, preceded by an opening statement with the aim of normalizing a certain behavior or feeling, which allows comparison of the representation that children have of the sibling relationship. The interviews were carried out by a graduate student. The Italian version of the SRI confirmed the three-factor structure of the original version and its reliability to assess qualitative aspects of the sibling relationship [67]. In this study, Cronbach’s alpha values of Affection, Conflict, and Rivalry dimensions were 0.73, 0.71, and 0.71, respectively.

### 2.3. Data Analysis

Data were analyzed using the Statistical Package for Social Sciences (SPSS), version 24 (2017). A post hoc power analysis was conducted using the software package, GPower 3.1.9.4. This post hoc analyses revealed the statistical power for this study was 0.98.

Descriptive statistics of quantitative data was performed for all dimensions. The normality of the dimensions was tested, using the directions of Curran and colleagues as criterion, which identified an accepted range for skewness from −2 to +2 and for kurtosis from −7 to +7 [68].

In order to verify whether the child and his/her sibling scored significantly different from each other in parenting style dimensions and sibling relationship we conducted, a t-test for paired sample separately for clinical and control group inserting the mean value of APQ and SRI dimensions reported by child and his/her sibling as paired variable.

Moreover, in order to verify whether there was a difference between clinical child (DBD), non-clinical siblings of DBD (S-DBD) and children of control group (CONTR), in parenting style, sibling relationship and psychopathological disorders, a series of one-way Analysis of CoVariance (ANCOVA) have been conducted inserting groups of children (DBD, S-DBD and CONTR) as factor, study variables (parenting style dimensions of APQ, sibling relationship dimensions of SRI, psychopathological disorders subscales of CBCL) as dependent variables, and gender as covariate. We decided to insert gender as covariate because wide literature showed that DBD is highly prevalent in males than females. For multiple comparisons, Bonferroni corrections were applied. Finally, post hoc tests were performed to evaluate how three groups differ in parenting style, sibling relationship and psychopathological disorders. The alpha level was set to *p* = 0.05 for all tests with confidence interval at 95%.

Finally, in order to verify whether there was a difference between clinical child (DBD), non-clinical siblings of DBD (S-DBD) and children of control group (CONTR), in distribution of presence and absence of CBCL-Dysregulation Profile a Chi-square was performed.

## 3. Results

### 3.1. Difference between Clinical and Control Group Families

Socio-demographic features of two groups are reported in Table 1.

T test and Chi square test were performed in order to verify if differences existed between clinical and control group in demographic variables. Results showed no difference in families of clinical and control group respect to parental marital status, number of children for family, maternal socio-economic level and paternal socio-economic level. All participants came from traditional families (mother-father), irrespectively by parental marital status reported (married or divorced).

Difference between offspring of clinical and control group.

Concerning offspring characteristics, data analysis showed no difference for age (clinical group M = 9.9; DS = 2; control group M = 9.8; DS = 2.2; t(120) = 0.34; n.s.), gender composition (% of males in clinical group = 49.4% versus control group = 50.6% Chi^2^(1) = 2.56; n.s.), and years of scholarship (clinical group M = 4.65; DS = 1.9 versus control group M = 4.62; DS = 2.1; t(120) = 0.08; n.s.).

All DBD children (27; 100%) received a clinical diagnosis of ODD with a prevalence of comorbidity with ADHD (19; 70.4%).

In line with the literature and characteristics of the disorder, the DBD group was mainly composed of males (25; 93%). A Chi-square test showed that there were differences in gender composition within clinical group sibling dyad (Chi^2^(1) = 9.63; *p* = 0.002) because males were 93% of DBD versus 56% of S-DBDs. On the contrary no difference has been found for gender composition in control group (56% of males in CONTR-1 versus 65% in CONTR-2; Chi^2^(1) = 0.553; n.s.).

### 3.2. Difference within Sibling Dyad

APQ and SRI mean scores reported by pair of siblings in clinical and control group are reported in Table 2.

A t-test for paired sample was performed separately for clinical and control group to verify whether sibling dyad differ in parenting style (APQ subscales), sibling relationship (SRI subscales). Results of t-test are reported in Table 2.

Concerning the APQ, no difference within the sibling dyad was found in the clinical group in positive parenting or corporal punishment. On the contrary, a difference emerged in negative parenting, with the parent reporting more negative parenting toward DBD child than S-DBD.

Moreover, no difference within the sibling dyad was found in the control group in positive parenting, negative parenting or corporal punishment.

Concerning to SRI no difference within sibling dyad was found in the clinical or control group in affection, conflict and rivalry.

### 3.3. Difference between Clinical, Non-Clinical Siblings and Control Group

APQ, SRI and CBCL subscale scores reported by DBD, S-DBD and CONTR are showed in Table 3.

A series of one-way ANCOVA inserting as dependent variables the APQ dimensions found no differences in positive parenting or corporal punishment among the three groups. On the contrary, a difference exists in negative parenting where DBD reported higher level of negative parenting than CONTR whilst adjusting for gender.

A series of one-way ANCOVA inserting as dependent variables the SRI dimensions showed that any difference exist in affection, conflict and rivalry between three groups whilst adjusting for gender. A series of one-way ANCOVAs inserting the CBCL dimensions as dependent variables showed that there were differences between the three groups in all scales and subscales of the CBCL questionnaire, except for somatic complaint, DSM somatic. DBD reported higher levels of disorder than S-DBD and CONTR children.

Results of ANCOVA are reported in Table 3.

### 3.4. DESR and DP Profile

The Chi-square test was performed using the distribution of DP among DBD, S-DBD and CONTR groups. Results showed that, among DBD children 9 (33.3%), presented DP, 11 (40.7%) DESR and 7 (26%) regular scores (above cut-off). Among S-DBD 2 children (7.4%) presented DP, 6 (22.2%) DESR and 19 (70.4%) regular score; in the CONTR group, none of children (0%) presented DP, one (1.5%) presented DESR and 67 (98.5%) regular scores. The difference of profile distribution among DBD and CONTR is significant (Chi^2^ (4) = 58.95; *p* = 0.000). In fact, while DBD presented higher prevalence of DP and DESR, while CONTR presented higher prevalence of regular scores.

## 4. Discussion

Literature has widely shown the role of the family in shaping DBD in children and adolescents. However, studies mainly focus on the impact of parenting style on DBD children [25,69,70], neglecting the presence of siblings. A perspective that takes into consideration parenting style effect on sibling dyads (including non-clinical ones) may identify risk and protective factors for psychological adjustment of the offspring from the same family, in order to better address preventive interventions.

In this framework, this study aimed to explore the parenting style and sibling relationship reported in sibling dyads of clinical families (where one child had been diagnosed with DBD and the other had not received any psychiatric diagnosis) compared to control families (in which there were two non-clinical siblings).

This study also aimed to compare parenting style, sibling relationship, and emotional and behavior problems reported in clinical child (DBD), non-clinical sibling (S-DBD), and non-clinical child of control families (CONTR). To our knowledge, this observational study is the first to explore and compare the parenting style reported toward two children (clinical and non-clinical) in families with DBDs and control families.

Our results show that there is higher differential and negative parenting in clinical families compared to control group families. Specifically, negative parenting is reported more often toward the DBD child compared to his/her sibling. On the contrary, control families reported a similar style across offspring. Results seem to confirm that even when siblings live in the same family context, they can experience different relationships with the same parents [37,71], and be exposed to different levels of harsh and supportive parenting, thus resulting in different adjustments [70,72]. Our study therefore confirms the link between higher negative parenting, which includes harsh parenting, coercive, controlling and punitive methods, and DBDs [25]. However, in contrast with previous research [23,25], in our study, the level of parental corporal punishment towards DBD did not differ from that reported by S-DBD and control sibling.

However, when we compared parenting styles adopted toward DBD, S-DBD, and control child, it emerged that, although lower levels of negative parenting were reported towards S-DBDs than their DBD siblings, these levels of negative parenting were higher than toward non-clinical siblings of the control group. The post-hoc analysis revealed that the main difference concerned DBDs who reported more negative parenting than CONTR. A possible explanation for this result may be the higher levels of parenting stress generated by the presence of a DBD child in clinical families [73,74], which induces parents to use negative parenting also toward the non-clinical sibling. Literature has widely shown an association between parenting stress and negative parenting [75,76,77,78], and between negative parenting and child difficulties, including externalizing behavior problems and adolescent deviance [79,80]. Thus, in line with the available literature [13,81,82,83,84], the use of negative parenting may be in part responsible for the severity of psychological disorders (as resulting from CBCL subscales scores). In our sample, DBD children, in a greater measure, and S-DBDs, in a lesser measure, reported higher externalizing problems (including rule-breaking behavior, aggressive behavior) than control siblings. It is possible that S-DBDs, despite being non-clinical siblings, could be at higher risk for externalizing disorders compared to non-clinical siblings growing up in non-clinical families, thus directly (personally) and/or indirectly (through observation of negative parenting style toward clinical sibling) exposed to negative parenting. In line with the literature, we could hypothesize that the clinical manifestation of DBDs (which includes irritability, aggression, and temper outburst) also constitutes a risk factor for the development of externalizing problems by siblings [81,82].

S-DBD children seem at higher risk for internalizing problems, such as anxious/depressed and withdrawn/depressed, compared to CONTR. In the case of internalizing problems and withdrawn/depressed scales, the level of problems reported by S-DBDs is not only higher than controls, but it is comparable to that reported by DBDs. A possible explanation is the parenting style. Several studies have reported an association between negative parenting (characterized by high levels of coercive control and negative emotionality) and internalizing more than externalizing symptoms [83]. In this case, it is possible that negative parenting reported toward DBDs and S-DBDs in clinical families increases the risk of internalizing problems in the offspring in general. Other explanations that must not be excluded are stressful life events, as well as negative and less supportive relationships with other family members, such as the father or other siblings, which may increase the possibility to develop internalizing problems. In literature, internalizing problems in DBDs have been described as predictors of persistence of externalizing problems in adolescents and/or of development of internalizing disorders over time [84,85]. Our research group has shown the efficacy of multimodal, CBT-based treatment involving DBD children and their parents, in improving internalizing problems in ODD patients by promoting effective and warm parenting, and supporting affect regulation and self–esteem in children [86]. We suppose that improvement in parenting style could have a positive effect in prevention of DBD sibling risk of internalizing problems.

Regarding emotional dysregulation profiles, the present study shows that about seven children in ten with DBDs manifest the clinical or severe profile of dysregulation (three in ten reported DP and four in ten reported DESR). This evidence is not surprising, because it is in line with the scientific literature that highlights the presence of poor emotional and behavioral self-regulation in children with DBDs alone or in comorbidity with ADHD and emotional problems [87,88,89].

An interesting result is the presence of a relevant percentage of DP in the S-DBD population; in fact, about three children in ten showed a clinical or severe profile (DESR and DP), while in the control group only 1.5% presented DESR and none reported DP. Our findings seem to suggest that not only clinical DBD children, but also non-clinical siblings of DBD families, show psychopathological vulnerability, being more prone to develop DESR and DP compared to the control group. The higher psychopathological vulnerability of S-DBD may be influenced by both environmental (such as parenting strategies) and genetic factors shared by the siblings in the clinical families. These data are worthy of attention, since the presence of DESR or DP could be considered both an indicator of clinical severity and a risk factor for developmental, emotional, or behavioral disorders from childhood to adulthood [63].

For this reason, during the diagnostic evaluation of the clinical child, the non-clinical sibling could also be involved, in order to identify or prevent the onset of emotional and behavioral problems early on. There is strong evidence that multimodal treatment programs for DBDs, usually including both youths and parents, have high efficacy in reducing externalizing behaviors and aggression in at-risk and clinic-referred youths [80,90,91,92]. Involvement of parents, aimed at reducing coercive strategies and promoting positive parenting styles, increases treatment efficacy [90,93], and could also have relevant positive effects for the siblings. It would be important to inform the families of children with DBDs about the potential psychopathological risks to the other children and provide intervention programs, such as parent training, to support positive parenting styles towards all offspring.

Regarding the sibling relationship, this study showed that there is no difference between sibling dyads for sibling relationship. In other words, both pairs of dyads reported the same sibling relationship. This is in line with the characteristics of the SRI. Moreover, no difference has been found for gender between DBD, S-DBD, and control children in affect, conflict, or rivalry. In other words, the sibling relationship in the clinical group is not characterized by more conflict compared to control group. It could be assumed that, due to the DBD clinical manifestation of disorder, the sibling relationship might be affected by greater levels of hostility; however, our results did not support this hypothesis.

Given the lack of difference between clinical and control groups, it seems that sibling relationship assumes a protective role in the clinical group. Thus, despite negative parenting reported toward DBDs and S-DBDs, the sibling relationship seems to be good enough for these children. This is a relevant aspect, because children spend more time with siblings than with parents, and within sibling dyads they develop social skills useful for future interactions with peers [94]. Like parent-child and peer relationships, sibling relationships provide an important socialization environment for children. Sibling relationships can be considered intense peer relationships, where children must learn to negotiate conflicts because they are unable to escape an aversive sibling relationship in the same manner as they would a peer [95].

According to our hypotheses, the results indicate a higher level of differential and negative parenting in clinical families, compared to control group families, confirming the link between higher negative parenting (i.e., harsh parenting, coercive, controlling and punitive methods), and DBDs and children with difficulties. Although negative parenting in clinical families is reported more often towards DBD children, DBD siblings showed higher levels than non-clinical siblings of the control group. The use of negative parenting seems to correlate with the severity of psychological disorders. In fact, in the clinical families, as resulting from CBCL subscale scores, higher externalizing problems (including rule-breaking behavior, aggressive behavior) were recorded not only in the DBD child, but also in the S-DBD, compared to control siblings (DBD > S-DBD > CONTR). Moreover, higher internalizing problems (including anxious/depressed and withdrawn/depressed) were recorded in DBD and S-DBD compared to control siblings (DBD = S-DBD > CONTR). In accordance with other studies [83], S-DBDs could be considered at higher risk for externalizing and internalizing problems than controls.

Contrary to our hypotheses, regarding sibling relationship, no differences between the two groups were found. In the clinical group, the sibling relationship seems to be good enough for these children, and in a preventive perspective, these data could highlight the importance of “protecting” the relationship between siblings in their growth path as a possible factor of resilience [50,95,96].

## 5. Strengths and Limitations of this Study

This is the first study that analyses the impact of parenting style on psychological disorders in DBD families (including both DBD clinical child and S-DBD non-clinical child) compared with non-clinical families. To this purpose, one of the main strengths of this study is the use of multiple source of information. In fact, we chose to include two siblings per family (who answered about sibling relationship), and one reference parent (who reported parenting style toward each child and psychological disorders shown by each child).

Although the sample size may appear quite small compared to other studies on sibling dyads [81], it must be noted that we chose to use strict inclusion criteria both for the clinical child (diagnosis of DBD according to DSM 5 instead of, for instance, the presence of externalizing behavior) and for sibling dyad (sibling dyads with maximum age spacing of four years in line with previous studies) [53]. The study presents a large effect size according to Cohen (1988) [97].

Second, we chose to consider only the mothers as reference parents, without involving fathers. We cannot exclude that paternal style may be different (i.e., more positive) than maternal, thus constituting a protective factor. The choice to consider the maternal report of parenting style comes from the consideration that Italian mothers play a major role both in and out of the home.

Finally, this is a cross sectional study. Thus, we cannot know if the parenting style changes over the time, including the level of psychological disorders expressed by the children.

## 6. Conclusions

In the developmental age, and more often in adolescence, there is physiological distancing in the sibling relationship, with a tendency to form their own groups of friends, who can often have interests in common and represent valid experiential contexts for prosocial purposes. In the case of families with DBDs, DBD children can dangerously tend to fit into groups of social marginalization that facilitate training in deviance with the adoption of highly antisocial values [98]. Maintaining a good sibling relationship could be a protective factor for DBDs against possible antisocial risks, and this aspect should be kept in mind, in cautionary and reinforcement messages to parents, and in therapeutic and preventive goals [99].

Finally, the involvement of parents in multimodal treatments to promote positive parenting could have positive effects not only for DBD children, but also for their siblings, in preventing internalizing and externalizing problems.

## Figures and Tables

**Table 1:** Comparison between Clinical and control group families in socio-demographic features.

	Clinical Group *n* = 27	Control Group *n* = 34	Statistics
Parental marital status			Chi^2^(1) = 1.23; n.s.
Married	20 (74%)	28 (82%)	
Divorced	7 (26%)	6 (18%)	
N children for family			Chi^2^(3) = 4.42; n.s.
2	15 (56%)	25 (74%)	
3	8 (30%)	6 (17%)	
4	3 (5%)	2 (6%)	
5	1 (4%)	1 (3%)	
Maternal SES			Chi^2^(3) = 6.66; n.s.
salariat	6 (22.2%)	9 (26.5%)	
intermediate	15 (55.6%)	22 (64.7%)	
working class	2 (7.4%)	0 (0%)	
unemployed	4 (14.8%)	3 (8.8%)	
Paternal SES			Chi^2^(3) = 6.18; n.s.
salariat	9 (33.3%)	8 (23.5%)	
intermediate	10 (37%)	19 (55.9%)	
working class	7 (25.9%)	7 (10.6%)	
unemployed	1 (3.7%)	0 (0%)	

Note: SES: Socio Economic Status.

**Table 2:** Mean (SD) reported by clinical sibling (DBD), non-clinical sibling of clinical family (S-DBD), control sibling 1 (CONTR-1) and control sibling 2 (CONTR-2) in APQ and SRI subscales. Results of t test are reported for both groups.

	Clinical Group		t(26)	*p*	Control Group		t(33)	*p*
	DBD	S-DBD			CONTR-1	CONTR-2		
APQ positive parenting	47.67 (4.60)	47.37 (4.65)	0.497	n.s.	46.65 (5.01)	47.23 (5.26)	−1.31	n.s.
APQ negative parenting	14.67 (4.22)	12.14 (4.14)	2.58	0.016	10.79 (3.59)	10.73 (3.54)	0.208	n.s.
APQ corporal punishment	4.41 (2.20)	4.33 (1.68)	0.311	n.s.	3.53 (0.896)	3.91 (1.28)	−1.97	n.s.
SRI affection	21.72 (6.96)	24.36 (5.74)	−1.51	n.s.	24.38 (3.29)	22.52 (4.86)	1.88	n.s.
SRI conflict	15.76 (4.09)	13.89 (3.55)	1.659	n.s.	13.41 (4.01)	14.08 (4.01)	−0.99	n.s.
SRI rivarly	8.04 (3.86)	7.88 (3.44)	0.148	n.s.	6.85 (3.40)	6.58 (2.54)	0.40	n.s.

**Table 3:** Mean (SD) reported by clinical sibling (DBD), non-clinical sibling of clinical family (S-DBD) and non-clinical sibling of control group (CONTR) in APQ, SRI and CBCL subscales. Results of one-way ANCOVA are reported.

	DBD	S-DBD	CONTR	F (2117)	*p*	Adjusted η^2^	Post-Hoc
	M (SD)	M (SD)	M (SD)				
APQ							
Positive parenting	47.67 (4.60)	47.37 (4.65)	47.10 (5.12)	0.182	n.s.	0.003	
Negative parenting	14.67 (4.22)	12.14 (4.14)	10.76 (3.54)	9.00	0.000	0.132	DBD > CONTR
Corporal punishment	4.41 (2.20)	4.33 (1.68)	3.72 (1.12)	2.44	n.s.	0.040	
SRI							
Affection	21.72 (6.96)	24.36 (5.74)	23.46 (4.23)	2.16	n.s.	0.036	
Conflict	15.76 (4.09)	13.89 (3.55)	13.75 (4.0)	2.91	n.s.	0.026	
Rivalry	8.04 (3.86)	7.88 (3.44)	6.69 (2.98)	2.50	n.s.	0.041	
CBCL							
Internalizing	62.56 (8.75)	56.62 (9.41)	49.82 (10.54)	13.57	0.000	0.188	DBD > S-DBD > CONTR
Externalizing	66.93 (8.45)	54.0 (8.29)	46.48 (7.65)	55.67	0.000	0.488	DBD > S-DBD > CONTR
Anxious/depressed	64.78 (8.87)	57.81 (6.90)	54.30 (5.74)	18.22	0.000	0.238	DBD > S-DBD > CONTR
Withdrawn/Depressed	60.93 (8.53)	57.88 (9.83)	54.28 (6.53)	5.70	0.004	0.089	DBD > S-DBD > CONTR
Somatic complain	56.15 (6.55)	56.29 (5.17)	54.60 (5.39)	1.05	n.s.	0.018	
Social prob.	62.44 (6.36)	55.96 (5.86)	53.49 (4.68)	23.75	0.000	0.289	DBD > S-DBD > CONTR
Thought prob.	61.33 (7.30)	54.92 (5.63)	53.39 (5.24)	15.72	0.000	0.212	DBD > S-DBD > CONTR
Attention prob.	67.15 (8.07)	60.07 (8.49)	53.15 (3.75)	46.98	0.000	0.445	DBD > S-DBD > CONTR
Rules breaking	61.81 (8.11)	55.62 (4.87)	52.03 (3.37)	32.72	0.000	0.359	DBD > S-DBD > CONTR
Aggressive behavior	69.33 (10.84)	56.0 (6.63)	52.12 (3.17)	60.06	0.000	0.507	DBD > S-DBD > CONTR
DSM Affective prob.	65.04 (8.06)	58.85 (7.66)	54.18 (5.98)	21.23	0.000	0.266	DBD > S-DBD > CONTR
DSM anxiety prob.	63.74 (8.01)	58.40 (7.74)	55.27 (6.08)	11.03	0.000	0.159	DBD > S-DBD > CONTR
DSM somatic prob.	54.56 (7.98)	54.22 (4.79)	54.10 (4.92)	0.03	n.s.	0.001	
DSM ADHD prob.	67.93 (9.32)	58.44 (8.93)	52.03 (3.04)	52.16	0.000	0.471	DBD > S-DBD > CONTR
DSM ODT	65.33 (8.99)	55.70 (5.29)	52.63 (3.25)	44.20	0.000	0.430	DBD > S-DBD > CONTR
DSM conduct prob.	65.19 (8.72)	54.92 (4.92)	51.69 (2.97)	56.81	0.000	0.493	DBD > S-DBD > CONTR

Note. Prob.: Problem; ODT: Oppositional Defiant Problems.

## Data Availability

Data are available by authors under reasonable request.

## References

[1] Coghill D. (2013). Editorial: Do clinical services need to take conduct disorder more seriously?. J. Child Psychol. Psychiatry.

[2] Polanczyk G.V., Salum G.A., Sugaya L.S., Caye A., Rohde L.A. (2015). Annual research review: A meta-analysis of the worldwide prevalence of mental disorders in children and adolescents. J. Child Psychol. Psychiatry.

[3] Patalay P., Gage S.H. (2019). Changes in millennial adolescent mental health and health-related behaviours over 10 years: A popula-tion cohort comparison study. Int. J. Epidemiol..

[4] American Psychiatric Association (2013). Diagnostic and Statistical Manual of Mental Disorders (DSM-5).

[5] Moffitt T.E., Arseneault L., Jaffee S.R., Kim-Cohen J., Koenen K.C., Odgers C.L., Slutske W.S., Viding E. (2008). Research review: DSM-V conduct disorder: Research needs for an evidence base. J. Child Psychol. Psychiatry.

[6] Burke J.D., Hipwell A.E., Loeber R. (2010). Dimensions of oppositional defiant disorder as predictors of depression and conduct disorder in preadolescent girls. J. Am. Acad. Child Adolesc. Psychiatry.

[7] Fairchild G., Hawes D.J., Frick P.J., Copeland W.E., Odgers C.L., Franke B., Freitag C.M., De Brito S.A. (2019). Conduct Disorder. Nat. Rev..

[8] Pardini D.A., Frick P.J., Moffitt T.E. (2010). Building an evidence base for DSM-5 conceptualizations of oppositional defiant disorder and conduct disorder: Introduction to the special section. J. Abnorm. Psychol..

[9] Rivenbark J.G., Odgers C.L., Caspi A., Harrington H., Hogan S., Houts R.M., Poulton R., Moffitt T.E. (2018). The high societal costs of childhood conduct problems: Evidence from administrative records up to age 38 in a longitudinal birth cohort. J. Child Psychol. Psychiatry.

[10] Karwatowska L., Russell S., Solmi F., De Stavola B.L., Jaffee S., Pingault J.-B., Viding E. (2020). Risk factors for disruptive behaviours: Protocol for a systematic review and meta-analysis of quasi-experimental evidence. BMJ Open.

[11] Jucksch V., Salbach-Andrae H., Lenz K., Goth K., Döpfner M., Poustka F., Holtmann M. (2011). Severe affective and behavioural dysregulation is associated with significant psychosocial adversity and impairment. Child Psychol. Psychiatry.

[12] Biederman J., Perry C.R., Day H., Goldin R.L., Spencer T., Faraone S.V., Surman C.B., Wozniak J. (2012). Severity of the aggression/anxiety-depression/attention (A-A-A) CBCL profile discriminates between different levels of deficits in emotional regulation in youth with ADHD. J. Dev. Behav. Pediatr..

[13] Masi G., Muratori P., Manfredi A., Lenzi F., Polidori L., Ruglioni L., Muratori F., Milone A. (2013). Response to treatments in youths with disruptive behavior disorders. Compr. Psychiatry.

[14] Masi G., Pisano S., Milone A., Muratori P. (2015). Child behavior checklist dysregulation profile in children with disruptive behavior disorders: A longitudinal study. J. Affect. Disord..

[15] Caspi A. (2000). The child is father of the man: Personality continuities from childhood to adulthood. J. Pers. Soc. Psychol..

[16] Cavanagh M., Quinn D., Duncan D., Graham T., Balbuena L.J. (2017). Oppositional Defiant Disorder Is Better Conceptualized as a Disorder of Emotional Regulation. J. Atten. Disord..

[17] Tremblay R.E. (2010). Developmental origins of disruptive behaviour problems: The ‘original sin’ hypothesis, epigenetics and their consequences for prevention. J. Child Psychol. Psychiatry.

[18] Pederson C.A., Fite P.J. (2014). The impact of parenting on the associations between child aggression subtypes and oppositional defiant disorder symptoms. Child Psychiatry Hum. Dev..

[19] Bornovalova M.A., Hicks B.M., Iacono W.G., McGue M. (2010). Familial transmission and heritability of childhood disruptive disorders. Am. J. Psychiatry.

[20] Caspi A., McClay J., Moffitt T.E., Mill J., Martin J., Craig I.W., Taylor A., Poulton R. (2002). Role of genotype in the cycle of violence in maltreated children. Science.

[21] Rowe R., Costello E.J., Angold A., Copeland W.E., Maughan B. (2010). Developmental pathways in oppositional defiant disorder and conduct disorder. J. Abnorm. Psychol..

[22] Tistarelli N., Fagnani C., Troianiello M., Stazi M.A., Adriani W. (2020). The nature and nurture of ADHD and its comorbidities: A narrative review on twin studies. Neurosci. Biobehav. Rev..

[23] Malmberg K., Wargelius H.L., Lichtenstein P., Oreland L., Larsson J.O. (2008). ADHD and Disruptive Behavior scores—Associations with MAO-A and 5-HTT genes and with platelet MAO-B activity in adolescents. BMC Psychiatry.

[24] Stormshak E.A., Bierman K.L., McMahon R.J., Lengua L.J. (2000). Parenting practices and child disruptive behavior problems in early elementary school. J. Clin. Child Psychol..

[25] Calkins S.D., Keane S.P. (2009). Developmental origins of early antisocial behavior. Dev. Psychopathol..

[26] Muratori P., Milone A., Nocentini A., Manfredi A., Polidori L., Ruglioni L., Lambruschi F., Masi G., Lochmann J.E. (2015). Maternal Depression and Parenting Practices Predict Treatment Outcome in Italian Children with Disruptive Behavior Disorder. J. Child Fam. Stud..

[27] Clark J.E., Frick P.J. (2016). Positive parenting and callous-unemotional traits: Their association with school behavior problems in young children. J. Clin. Child Adolesc. Psychol..

[28] Waller R., Gardner F., Hyde L.W. (2013). What are the associations between parenting, callous–unemotional traits, and antisocial behavior in youth? A systematic review of evidence. Clin. Psychol. Rev..

[29] Waller R., Gardner F., Viding E., Shaw D.S., Dishion T.J., Wilson M.N., Hyde L.W. (2014). Bidirectional associations between pa-rental warmth, callous unemotional behavior, and behavior problems in high-risk preschoolers. J. Abnorm. Child Psychol..

[30] Muratori P., Lochman J.E., Lai E., Milone A., Nocentini A., Pisano S., Righini E., Masi G. (2016). Which dimension of parenting predicts the change of callous unemotional traits in children with disruptive behavior disorder?. Compr. Psychiatry.

[31] Patterson G.R., Reid J.B., Patterson G.R., Snyder J. (2002). The early development of coercive family process. Antisocial Behavior in Children and Adolescents: A Developmental Analysis and Model for Intervention.

[32] Pettit G.S., Arsiwalla D.D. (2008). Commentary on special section on “bidirectional parent–child relationships”: The continuing evolution of dynamic, transactional models of parenting and youth behavior problems. J. Child Psychol. Psychiatry.

[33] Deater-Deckard K. (2001). Annotation: Recent research examining the role of peer relationships in the development of psychopathology. J. Child Psychol. Psychiatry.

[34] Richmond M.K., Stocker C.M., Rienks S.L. (2005). Longitudinal associations between sibling relationship quality, parental differential treatment, and children’s adjustment. J. Fam. Psychol..

[35] Asbury K., Dunn J., Plomin R. (2006). Birthweight-discordance and differences in early parenting relate to monozygotic twin differences in behaviour problems and academic achievement at age 7. Dev. Sci..

[36] Asbury K., Dunn J., Plomin R. (2006). The use of discordant MZ twins to generate hypotheses regarding non-shared environmental influence on anxiety in middle childhood. Soc. Dev..

[37] Burt S.A., McGue M., Iacono W.G., Krueger R.F. (2006). Differential Parent-child Relationships and Adolescent Externalizing Symptoms: Cross-Lagged Analyses within a Twin Differences Design. Dev. Psychol..

[38] Mullineaux P.Y., Deater-Deckard K., Petrill S.A., Thompson L.A. (2009). Parenting and child behaviour problems: A longitudinal analysis of non-shared environment. Infant Child Dev..

[39] Ilomäki E., Viilo K., Hakko H., Marttunen M., Mäkikyrö T., Räsänen P. (2006). Familial risks, conduct disorder and violence: A Finnish study of 278 adolescent boys and girls. Eur. Child Adolesc. Psychiatry.

[40] Feinberg M.E., Hetherington E.M. (2001). Differential parenting as a within-family variable. J. Fam. Psychol..

[41] Stocker C.M., McHale S. (1992). The nature and family correlates of preadolescents’ perceptions of their sibling relationships. J. Soc. Pers. Relat..

[42] Dalsgaard S., Ostergaard S.D., Leckman J.F., Mortensen P.B., Pedersen M.G. (2015). Mortality in children, adolescents, and adults with attention deficit hyperactivity disorder: A nationwide cohort study. Lancet.

[43] Maibing C.F., Pedersen C.B., Benros M.E., Mortensen P.B., Dalsgaard S., Nordentoft M. (2015). Risk of schizophrenia increases after all child and adolescent psychiatric disorders: A nationwide study. Schizophr. Bull..

[44] Erskine H.E., Ferrari A.J., Polanczyk G.V., Moffitt T.E., Murray C.J., Vos T., Whiteford H.A., Scott J.G. (2014). The global burden of conduct disorder and attention-deficit/hyperactivity disorder in 2010. J. Child Psychol. Psychiatry.

[45] Carrà E., Marta E. (1995). Relazioni Familiari e Adolescenza [Family Relationships and Adolescence].

[46] Scabini E. (2000). Parent–child relationships in Italian families: Connectedness and autonomy in the transition to adulthood. Psicol. Teor. Pesqui..

[47] McArdle P., Wiegersma A., Gilvarry E., Kolte B., McCarthy S., Fitzgerald M., Brinkley A., Blom M., Stoeckel I., Pierolini A. (2002). European adolescent substance use: The roles of family structure, function and gender. Addiction.

[48] Smorti M., Guarnieri S., Ingoglia S. (2014). The parental bond, resistance to peer influence, and risky driving in adolescence. Transp. Res. Part F Traffic Psychol. Behav..

[49] Brody G.H., Stoneman Z., McCoy J.K. (1992). Associations of maternal and paternal direct and differential behavior with sibling relationships: Contemporaneous and longitudinal analyses. Child Dev..

[50] McHale S.M., Updegraff K.A., Whiteman S.D. (2012). Sibling Relationships and Influences in Childhood and Adolescence. J. Marriage Fam..

[51] Singer A.T., Weinstein R.S. (2000). Differential parental treatment predicts achievement and self-perceptions in two cultural contexts. J. Fam. Psychol..

[52] Shanahan L., McHale S.M., Crouter A.C., Osgood D. (2008). Linkages between parents’ differential treatment, youth depressive symptoms, and sibling relationships. J. Marriage Fam..

[53] Tamrouti-Makkink I.D., Dubas J.S., Gerris J.R., van Aken M.A. (2004). The relation between the absolute level of parenting and differential parental treatment with adolescent siblings’ adjustment. J. Child Psychol. Psychiatry.

[54] Kaufman J., Birmaher B., Brent D., Rao U., Flynn C., Moreci P., Williamson D., Ryan N. (1997). Schedule for affective disorders and schizophrenia for school-age children-present and lifetime version (K-SADS-PL): Initial reliability and validity data. J. Am. Acad. Child Adolesc. Psychiatry.

[55] Wechsler D. (2003). WISC-IV Technical and Interpretive Manual.

[56] Achenbach T., Rescorla L. (2001). Manual for the ASEBA School-Age Forms & Profiles.

[57] Achenbach T.M., Dumenci L., Rescorla L.A. (2003). DSM-oriented and empirically based approaches to constructing scales from the same item pools. J. Clin. Child Adolesc. Psychol..

[58] Frigerio A., Vanzin L., Pastore V., Nobile M., Giorda R., Marino C., Molteni M., Rucci P., Ammaniti M., Lucarelli L. (2006). The Italian preadolescent mental health project (PrISMA): Rationale and methods. Int. J. Methods Psychiatr. Res..

[59] Masi G., Muratori P., Manfredi A., Pisano S., Milone A. (2015). Child Behaviour Checklist emotional dysregulation profiles in youth with disruptive behaviour disorders: Clinical correlates and treatment implications. Psychiatry Res..

[60] Muratori P., Pisano S., Milone A., Masi G. (2017). Is emotional dysregulation a risk indicator for auto-aggression behaviors in adolescents with oppositional defiant disorder?. J. Affect. Disord..

[61] Biederman J., Petty C.R., Monuteaux M.C., Evans M., Parcell T., Faraone S.V., Wozniak J. (2009). The child behavior checklist-pediatric bipolar disorder profile predicts a subsequent diagnosis of bipolar disorder and associated impairments in ADHD youth growing up: A longitudinal analysis. J. Clin. Psychiatry.

[62] Ayer L., Althoff R., Ivanova M., Rettew D., Waxler E., Sulman J., Hudziak J. (2009). Child Behavior Checklist Juvenile Bipolar Disorder (CBCL-JBD) and CBCL Posttraumatic Stress Problems (CBCL-PTSP) scales are measures of a single dysregulatory syndrome. J. Child Psychol. Psychiatry.

[63] Holtmann M., Buchmann A.F., Esser G., Schmidt M.H., Banaschewski T., Laucht M. (2011). The Child Behavior Checklist-Dysregulation Profile predicts substance use, suicidality, and functional impairment: A longitudinal analysis. J. Child Psychol. Psychiatry.

[64] Sheldon K.K., Frick P.J., Wootton J. (1996). Assessment of parenting practices in families of elementary school-age children. J. Clin. Child Psychol.

[65] Esposito A., Servera M., Garcia-Banda G., Del Giudice E. (2016). Factor analysis of the Italian version of the Alabama Parenting Questionnaire in a community sample. J. Child. Fam. Stud..

[66] Stocker C.M., Lanthier R.P., Furman W. (1997). Sibling relationships in early adulthood. J. Fam. Psychol..

[67] Lecce S., De Bernart D., Vezzani C., Pinto G., Primi C. (2011). Measuring the quality of the sibling relationship during middle childhood: The psychometric properties of the Sibling Relationship Inventory. Eur. J. Dev. Psychol..

[68] Curran P.J., West S.G., Finch J.F. (1996). The robustness of test statistics to non normality and specification error in confirmatory factor analysis. Psychol. Methods.

[69] Burke J.D., Pardini D.A., Loeber R. (2008). Reciprocal relationships between parenting behavior and disruptive psychopathology from childhood through adolescence. J. Abnorm. Child Psychol..

[70] Tung I., Lee S.S. (2014). Negative parenting behavior and childhood oppositional defiant disorder: Differential moderation by positive and negative peer regard. Aggress. Behav..

[71] Dunn J.F., Stocker C., Plomin R. (1990). Nonshared experiences within the family: Correlates of behavioral problems in middle childhood. Dev. Psychopathol..

[72] Jeannin R., Van Leeuwen K. (2015). Associations between direct and indirect perceptions of parental differential treatment and child socio-emotional adaptation. J. Child Fam. Stud..

[73] Hart M.S., Kelley M.L. (2006). Fathers’ and mothers’ work and family issues as related to internalizing and externalizing behavior of children attending day dare. J. Fam. Stress.

[74] Spratt E.G., Saylor C.F., Macias M.M. (2007). Assessing parenting stress in multiple samples of children with special needs (CSN). Fam. Syst. Health.

[75] Abidin R.R. (1986). Parenting Stress Index.

[76] Deater-Deckard K., Scarr S. (1996). Parenting stress among dual-earner mothers and fathers: Are there gender differences?. J. Fam. Psychol..

[77] Le Y., Fredman S.J., Feinberg M.E. (2017). Parenting stress mediates the association between negative affectivity and harsh parenting: A longitudinal dyadic analysis. J. Fam. Psychol..

[78] Mak M.C.K., Yin L., Li M., Cheung R.Y.-H., Oon P.-T. (2020). The Relation between Parenting Stress and Child Behavior Problems: Negative Parenting Styles as Mediator. J. Child Fam. Stud..

[79] Qi C.H., Kaiser A.P. (2003). Behavior problems of preschool children from low-income families: Review of the literature. Top. Early Child. Spec. Educ..

[80] Bradley R.H., Corwyn R.F. (2007). Externalizing problems in fifth grade: Relations with productive activity, maternal sensitivity, and harsh parenting from infancy through middle childhood. Dev. Psychol..

[81] Craine J.L., Tanaka T.A., Nishina A., Conger K.J. (2009). Understanding adolescent delinquency: The role of older siblings’ delinquency and popularity with peers. Merrill-Palmer Q..

[82] Defoe I.N., Keijsers L., Hawk S.T., Branje S., Dubas J.S., Buist K., Frijns T., van Aken M.A., Koot H.M., van Lier P.A. (2013). Siblings versus parents and friends: Longitudinal linkages to adolescent externalizing problems. J. Child Psychol. Psychiatry.

[83] Pinquart M. (2017). Associations of parenting dimensions and styles with externalizing problems of children and adolescents: An updated meta-analysis. Dev. Psychol..

[84] Jacobs R.H., Becker-Weidman E.G., Reinecke M.A., Jordan N., Silva S.G., Rohde P., March J.S. (2010). Treating depression and oppositional behavior in adolescents. J Clin. Child Adolesc. Psychol..

[85] Nock M.K., Kazdin A.E., Hiripi E., Kessler R.C. (2007). Lifetime prevalence, correlates and persistence of oppositional defiant dis-order: Results from the National Comorbidity Survey Replication. J. Child Psychol. Psychiatry.

[86] Masi G., Milone A., Paciello M., Lenzi F., Muratori P., Manfredi A., Polidoi L., Ruglioni L., Lochman J.E., Muratori F. (2014). Effi-cacy of a multimodal treatment for disruptive behavior disorders in children and adolescents: Focus on internalizing problems. Psychiatry Res..

[87] Muratori P., Milone A., Manfredi A., Polidori L., Ruglioni L., Lambruschi F., Masi G., Lochman J.E. (2017). Evaluation of Improvement in Externalizing Behaviors and Callous-Unemotional Traits in Children with Disruptive Behavior Disorder: A 1-Year Follow Up Clinic-Based Study. Adm. Policy Ment. Health Ment. Health Serv. Res..

[88] Aitken M., Battaglia M., Marino C., Mahendran N., Andrade B.F. (2019). Clinical utility of the CBCL Dysregulation Profile in children with disruptive behavior. J. Affect. Disord..

[89] Spencer T.J., Faraone S.V., Surman C.B.H., Petty C., Clarke A., Batchelder H., Wozniak J. (2011). Towards defining deficient emo-tional self-regulation in youth with attention deficit hyperactivity disorder using the Child Behavior Checklist: A controlled study. Postgrad. Med. J..

[90] Eyberg S.M., Nelson M.M., Boggs S.R. (2008). Evidence-based psychosocial treatments for children and adolescents with disruptive behavior. J. Clin. Child Adolesc. Psychol..

[91] Lochman J.E., Powell N.P., Boxmeyer C.L., Jimenez-Camargo L. (2011). Cognitive behavioral therapy for externalizing disorders in children and adolescents. Child Adolesc. Psychiatr. Clin. N. Am..

[92] Kochanska G., Kim S., Boldt L.J., Yoon J.E. (2013). Children’s callous-unemotional traits moderate links between their positive relationships with parents at preschool age and externalizing behavior problems at early school age. J. Child Psychol. Psychiatry Allied Discip..

[93] Garland A.F., Hawley K.M., Brookman-Frazee L., Hurlburt M.S. (2008). Identifying common elements of evidence-based psycho-social treatments for children’s disruptive behaviour problems. J. Am. Acad. Child Adolesc. Psychiatry.

[94] Smorti M., Ponti L. (2018). How Does Sibling Relationship Affect Children’s Prosocial Behaviors and Best Friend Relationship Quality?. J. Fam. Issues.

[95] Newman J. (1994). Conflict and friendship in sibling relationships: A review. Child Study J..

[96] Gass K., Jenkins J., Dunn J. (2007). Are sibling relationships protective? A longitudinal study. J. Child Psychol. Psychiatry.

[97] Cohen J. (1988). Statistical Power Analysis for the Behavioral Sciences.

[98] Chen D., Drabick D.A.G., Burgers D.E. (2015). A Developmental Perspective on Peer Rejection, Deviant Peer Affiliation, and Conduct Problems among Youth. Child Psychiatry Hum. Dev..

[99] Feinberg M.E., Solmeyer A.R., McHale S.M. (2012). The Third Rail of Family Systems: Sibling Relationships, Mental and Behavioral Health, and Preventive Intervention in Childhood and Adolescence. Clin. Child Fam. Psychol. Rev..

